# Characterization of tubular liquid crystal structure in embryonic stem cell derived embryoid bodies

**DOI:** 10.1186/s13578-016-0130-6

**Published:** 2017-01-03

**Authors:** MengMeng Xu, Odell D. Jones, Liyang Wang, Xin Zhou, Harry G. Davis, Joseph L. Bryant, Jianjie Ma, Willian B. Isaacs, Xuehong Xu

**Affiliations:** 1 Department of Pharmacology, Duke University Medical Center, Durham, NC 27708 USA; 2 University of Pennsylvania ULAR, Philadelphia, PA 19144 USA; 3 The Laboratory of Cell Genetics and Developmental Biology (CGDB), Shaanxi Normal University College of Life Sciences, Xi’an, 710062 Shaanxi People’s Republic of China; 4 Institute of Human Virology, University of Maryland School of Medicine, Baltimore, MD USA; 5 Ohio State University School of Medicine, Columbus, OH 43210 USA; 6 Johns Hopkins School of Medicine, Baltimore, MD 21287 USA

**Keywords:** Liquid crystal, Phase transition, Embryoid body, Embryonic stem cell

## Abstract

**Background:**

Massive liquid crystal droplets have been found during embryonic development in more than twenty different tissues and organs, including the liver, brain and kidney. Liquid crystal deposits have also been identified in multiple human pathologies, including vascular disease, liver dysfunction, age-related macular degeneration, and other chronic illnesses. Despite the involvement of liquid crystals in such a large number of human processes, this phenomenon is poorly understood and there are no in vitro systems to further examine the function of liquid crystals in biology.

**Results:**

We report the presence of tubular birefringent structures in embryoid bodies (EBs) differentiated in culture. These birefringent tubular structures initiate at the EB surface and penetrated the cortex at a variety of depths. Under crossed polarized light, these tubules are seen as a collection of birefringent Maltese crosses and tubules with birefringent walls and a non-birefringent lumen. The fluidity of these birefringent structures under pressure application led to elongation and widening, which was partially recoverable with pressure release. These birefringent structures also displayed heat triggered phase transition from liquid crystal to isotropic status that is partially recoverable with return to ambient temperature. These pressure and temperature triggered changes confirm the birefringent structures as liquid crystals. The first report of liquid crystal so early in development.

**Conclusion:**

The structure of the liquid crystal tubule network we observed distributed throughout the differentiated embryoid bodies may function as a transportation network for nutrients and metabolic waste during EB growth, and act as a precursor to the vascular system. This observation not only reveals the involvement of liquid crystals earlier than previously known, but also provides a method for studying liquid crystals in vitro.

**Electronic supplementary material:**

The online version of this article (doi:10.1186/s13578-016-0130-6) contains supplementary material, which is available to authorized users.

## Dear editor

Liquid crystals have been reported in more than 20 embryonic tissues and organs, including the developing liver, yolk sac, and blood [1–6]. Further studies have proven that hepatic liquid crystals are omnipresent in all vertebrate liver during development [5, 7–13]. Despite prevalence in organs systems and across species, the function of liquid crystals during animal development remains unknown. To study liquid crystals earlier in the developmental timeline, we developed embryoid bodies (EBs) aggregated from pluripotent cells derived from embryonic stem (ES) cells or reprogrammed from adult epithelial skin cells or induced pluripotent stem (iPS) cells [14–16]. Since EBs are made up of a large variety of differentiated cell types growing in a three dimensional space, they are considered a valuable model system for investigating cellular and molecular interactions during the earliest stages of development. Because EB cells are precursors to the full spectrum of cell types needed to repair damaged tissue, better understanding of them offers great promise in clinical application. The most interesting of these applications is the potential of the EB system as a drug screening platform for multiple diseases, including heart disease, cancer, and other chronic illnesses [1, 17–21].

In this study, we report the unexpected presence of liquid crystals in EBs aggregated from embryonic stem cells. Systematic characterization of these birefringent liquid crystals show these complexes to be distributed throughout the EB as a network of tubules. The contours of these tubular liquid crystal tubes resemble the vascular network within the human body, linking the outside environment to the EB interior. Due to this structure, we postulate that liquid crystals may function as a transportation network for nutrients and metabolic waste during EB growth. By revealing the possible function of liquid crystals as the “highway” of the EB cluster, our findings provide valuable information towards understanding the function of liquid crystals in animal development and reveal their role in establishing a possible mode of communication between a tissue and the external environment.

## Birefringent droplets and tubular structure complex form in differentiated EB spheres

To investigate the function of liquid crystals, we utilized the human embryonic stem cell H9 and induced pluripotent stem cells (iPS DF19-9-7T) to carry out embryoid body aggregation. Stem cells were seeded and maintained in mTeSR-1 media as previously described (Additional file 1: Figure S1). The ES cell clumps were cultured on low attachment Petri dishes coated with 0.1% gelatin. H9 and DF19-9-7T were maintained on culture plates coated with Matrigel (StemCell Technology) and cultured in mTeSR-1 media. After cell clumps have aggregated, EBs were allowed to grow for 8 weeks and form differentiated spheres. These EB differentiated spheres were then harvested and cryosectioned at the thickness of 10 μm for observations under polarization microscope. Oct-3/4 expression in these samples was detected using cytochemistry and RT-PCR conducted as detailed in Additional file 1: Figure S1.

**Fig. 1:**
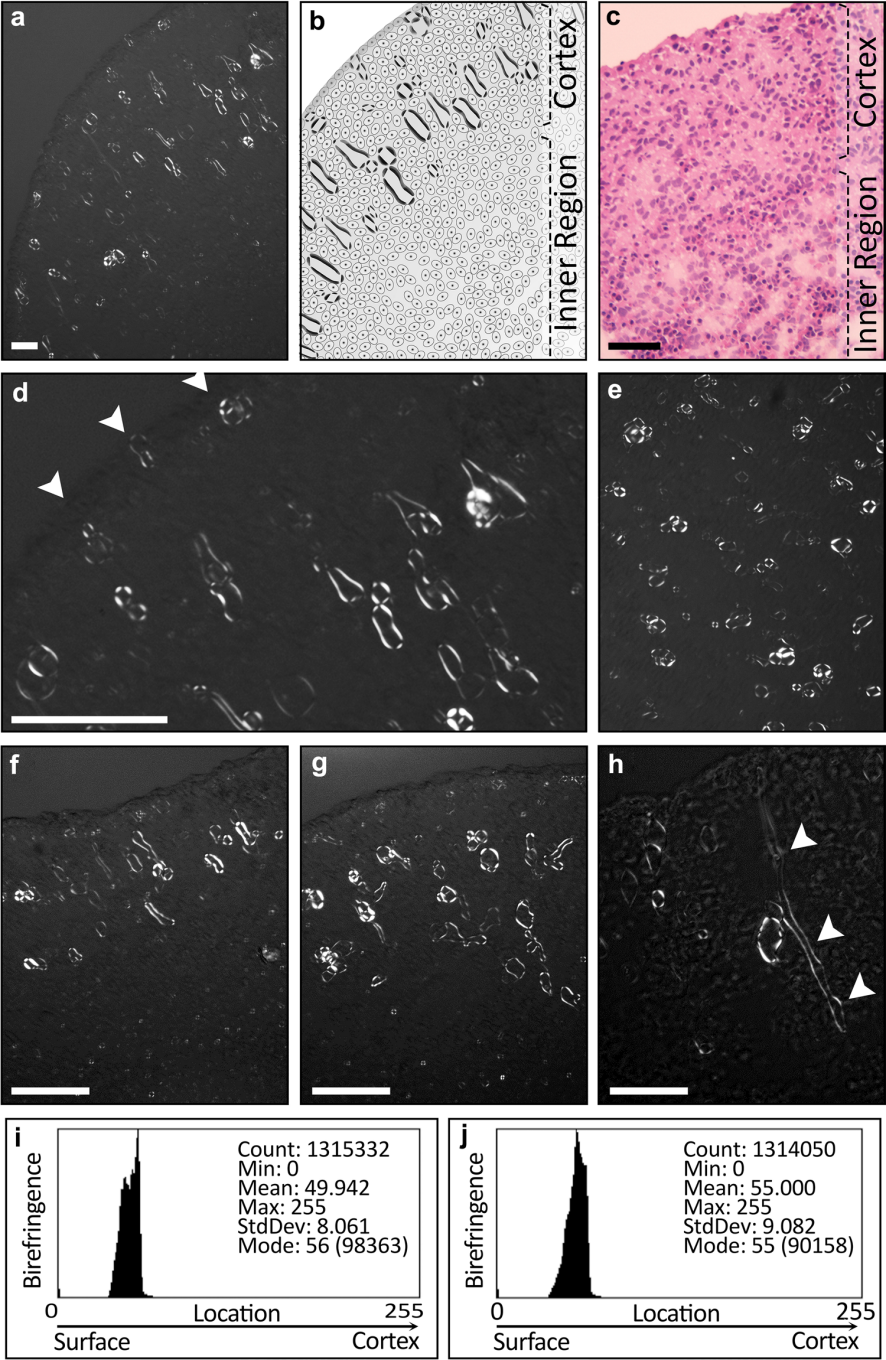
The birefringent structures in the embryoid bodies derived from H9 and induced iPS DF19-9-7T) at 8-weeks after differentiation. The liquid crystal birefringence distribution in the cortex region (CR) of the EB in (**a**) and sketched (**b**) compared to H&E histology (**c**). The structures of the birefringence are mainly tubular or droplet shaped with the wider-ends oriented towards the center of the EB (**d**). *Arrows* in **d** denote the individual ~35 μm spaced birefringent clusters from which liquid crystal tubules originate on the EB surface. Cross sectional cut exposing the tubular structures (**e**). The birefringence distributions in two representative cortex areas of an EB (**f**, **g**) and their respective quantifications (**i**, **j**). Representative image showing the trajectory of a tubule penetrating to the EB core when cut along the longitudinal axis (**h**). The *scale bars* are 60 μm in **a** and **c** and 300 μm in **d**–**h**

**Fig. 2:**
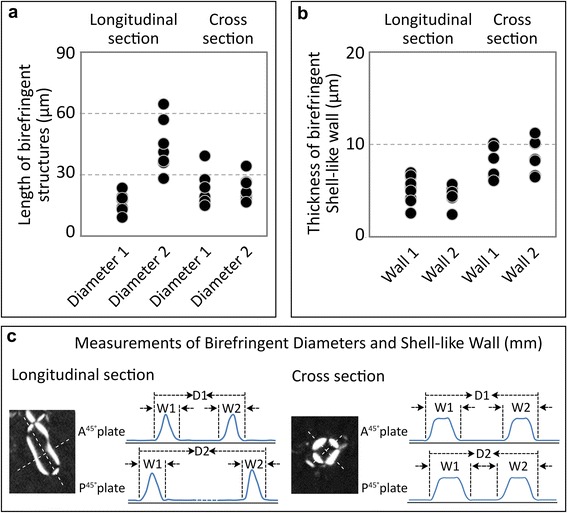
Distribution of the longitudinal and cross sections of the EB tubular liquid crystal structures. Quantified measurements of longitudinal and cross sectional diameter lengths (**a**). Quantified thickness measurements of the shell-like wall (**b**). All measurements were obtained from birefringence of multiple cross sectional and longitudinal sections (For longitudinal diameters *D1* and *D2*, n = 24; For cross diameters *D1* and *D2*, n = 24; For longitudinal and cross sections, wall-thickness *W1* and *W*2, n = 23). The diameter measurement is obtained from the distance between the outer edges of both sides of the birefringence along the long and short axis. Wall measurements are defined as the average width of the birefringence on each side of the axis (**c**)

Observation of EB spheres under polarization microscope revealed unexpected birefringent structures not identifiable through hematoxylin and eosin (H&E) histology (Fig. 1a–c) with standard method [22–25]. The overall shapes of these birefringent structures were droplet-like and tubular. They originated at the EB sphere surface and tunneled deeper into cortex, with the highest density of tubes observed near the EB periphery. These liquid crystal complexes begin with clusters of two or three birefringent droplets located just within the outer surface and distributed ~35 μm apart (Fig. 1d). Tubules from these birefringent clusters penetrate towards the cortex to a variety of depths (Fig. 1a–h). While most of these structures terminate before reaching the center of the EB (Fig. 1f–j), some birefringent tubules penetrate the entire cell cluster until reaching the core of the sphere (Fig. 1h). Under crossed polarized light, these liquid crystal tubules present as a collection of birefringent droplets in the shape of Maltese crosses and tubules with birefringent shell-like walls and a non-birefringent lumen (Fig. 1e–h). The Maltese crosses are cross sectioned images of the tubules while the clearly tubular birefringence are these same structures caught in longitudinal section. The lengths of these tubules vary from 40 μm to 2 mm, while the diameters consistently measured between 20 and 60 μm (Fig. 2a, c). The thickness of the tubular wall, as defined by the birefringence of the “shell”, measured at 4 and 9 μm for the longitudinal and cross-sectional images, respectively (Fig. 2b, c). This difference in thickness, as defined by birefringence, is logical given that cross sectional slices along a tube would lead to a thicker deposit of birefringent material, which in turn causes the brighter birefringence that measures as a thicker wall.

## Fluidity of birefringent structures in differentiated EBs

To characterize liquid crystal fluidity of the birefringent structures, pressure-recovery experiments were conducted on the EB smear samples. Following previously described protocol, pressure was applied evenly on the cover-glass with a rubber applicator and measurements of its effect on the birefringent structures were made between crossed polarized light and analyzed via ImageJ. Documentations on distances and density were based on birefringence of the Maltese cross and tubule structure [6].

**Fig. 3:**
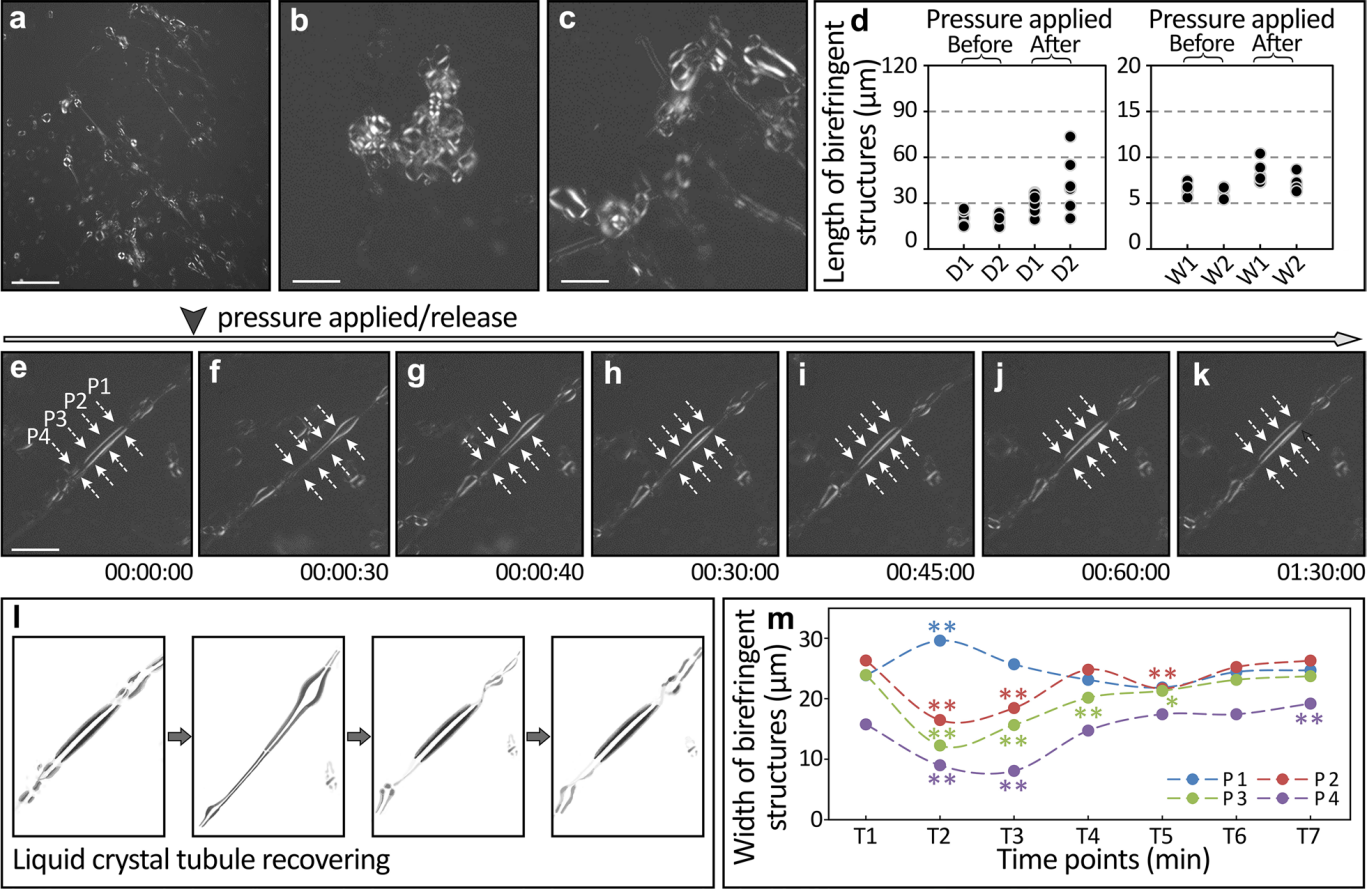
Fluidity characterization of the liquid crystal structures in embryoid bodies derived from H9 and induced iPS DF19-9-7T using the pressure application and release approach. The structures exhibited elongation after pressure application (**a**). The birefringent complex structures were spread and flattened (**c**) compared to the structures prior to pressure application (**b**) in the same view. This change in width was insignificant (**d**). Time elapse images of the birefringent tubular structure before (**e**) and after pressure application (**f**–**k**) documents the structural distortion and recovery. Higher magnification images of liquid crystal tube recovery and quantifications of width distortion and recovery in several different tubules are shown (**l** and **m**, respectively). Statistical significance of tubule width changes in *T2*–*T7* of *panel* **m** references time point 1 (*T1*) of respective color-coded tubules *P1*–*P4*. *p < 0.05 and **p < 0.01 in *panel* **m**. *Scale bars* 300 μm in (**a)**; 60 μm in **b** and **c**; 120 μm in **e**–**k**

After pressure was applied, the birefringent structures were distorted into elongated tubules (Fig. 3a, d). Compared to before the pressure application (Fig. 3b), flattening of the liquid crystal tubules under pressure lead to an enlarged birefringent complex (from Fig. 3c). This distortion in tubular length and width is quantified in Fig. 3d. Time lapse images from a single tubule in Fig. 3e–m demonstrate the tubule distortion and partial recovery after pressure application. This recovery requires approximately 60–90 min, depending on the size of the birefringent tubule. The flexibility of birefringent tubules leads to a variety of recovery shapes and processes. From the movement and characteristics documented above, we conclude that the EBs birefringent tubular structures have the fluidity and birefringence activity typical of liquid crystal.

## Liquid crystal characteristics confirmed in EB structures during temperature phase transition experiments

The droplet and tubular liquid crystals (D/T-LC) identified in differentiated EB spheres have thus far exhibited the birefringence and pressure recovery characteristics typical of true liquid crystals. Here we demonstrate that the birefringent D/T-LC structures also conform to the temperature-sensitive phase transitional properties typical of liquid crystals.

**Fig. 4:**
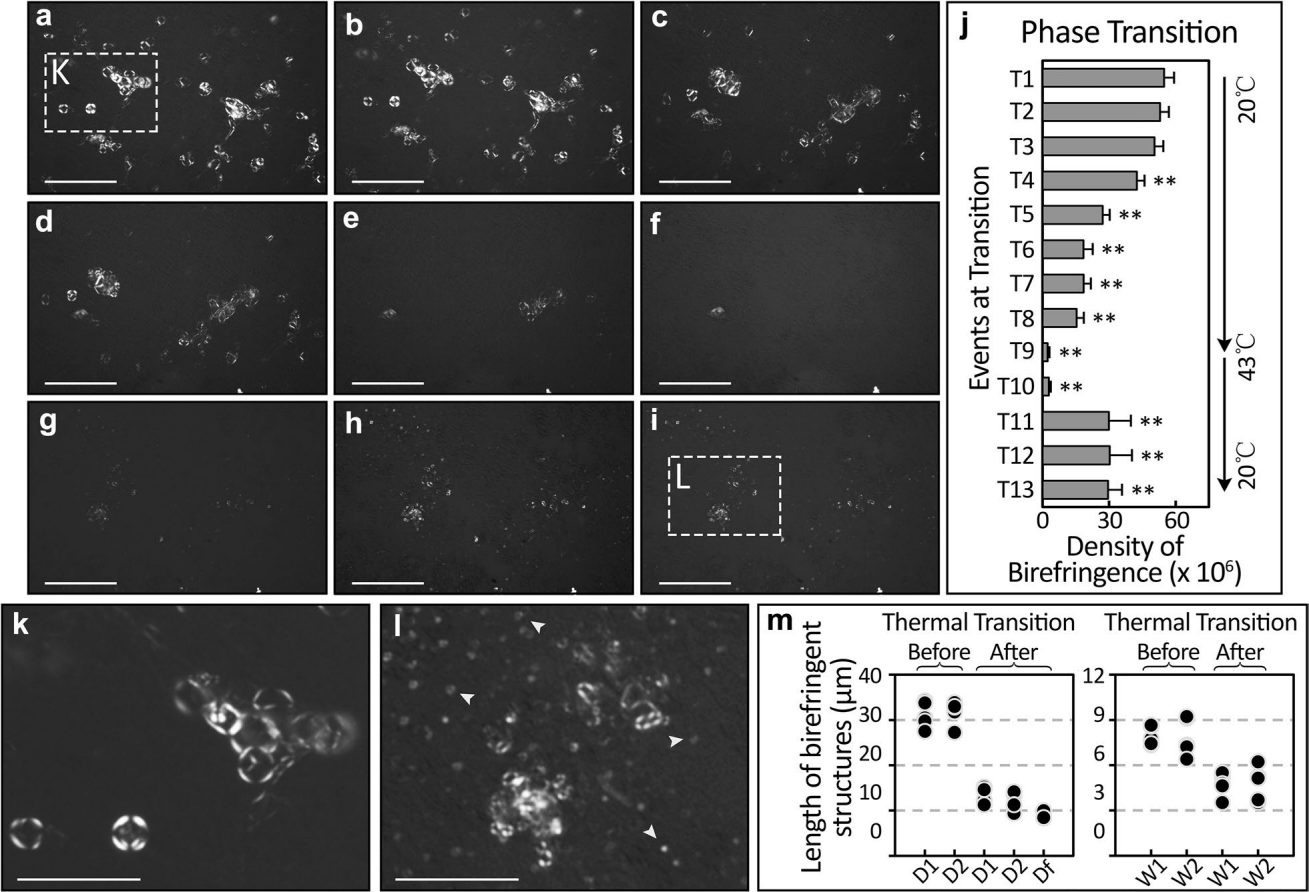
Time-lapse recordings of temperature dependent phase transition of embryoid body birefringent liquid crystals. Birefringence of liquid crystals distributed in the embryoid body is lost as the crystals assume isotropic status with temperature increase to 43 °C (**a**–**f**). There is incomplete recovery of the birefringent structure as the thermostage resumed room temperature (**h**, **i**). This change in birefringence following temperature change and time progression is quantifiable (**j**). There are no significant differences among birefringence density of *T1–T3*, but extremely significant difference in *T4–T13* based on references *T1*. Magnifications of the liquid–crystal structures before and after temperature transition highlight the incomplete resumption of the original structure (**k**, **l**, respectively). The decrease in size of EB liquid crystal structures is quantified by the significantly smaller diameters Maltese’s cross (*D1* before and *D1* after, p = 0.000003; *D2* before and *D2* after, p = 0.000001) and tubular structures (*W1* before and *W1* after, p = 0.000083; *W2* before and *W1* after, p = 0.003474) is quantified (M). *p < 0.05 and **p < 0.01 in **j**. *Scale bars* are 300 μm in (**a**–**i)**; 100 μm in (**k**, **l)**

Using temperature alternations generated by a thermo-stage, we show that D/T-LC structures phase transition from liquid crystal status to isotropic status when the temperature is increased to 43 °C. This process is partially reversible when ambient temperature is restored. This transition is seen as disappearing birefringence with temperature increase (Fig. 4a–g) and partial birefringence recovery (Fig. 4h, i). This effect is dramatic and quantifiable in Fig. 4j. A higher magnification of the D/T-LC structures before and after temperature-transition is shown to demonstrate the incomplete recovery of liquid crystal structure (Fig. 4k, l). Quantification of birefringent structures show that recovery from the isotropic state results in significant smaller Maltese crosses (Fig. 4m). Evenly distributed fragments of birefringent droplet were also documented in these post-temperature change EB (indicated by arrow heads).

The tubular liquid crystal structure is an extremely flexible entity capable of assuming different forms. Temperature is key to increasing the malleability of liquid crystals. When the temperature is raised, the tubular structure breaks into fragments. As seen in our experiments, these fragmented liquid crystal tubules retain their liquid crystal optical properties, but take the form of easily manipulated droplets (Fig. 4l, m). This thermal triggered phase transition and structural flexibility is typical property of liquid crystals and can be easily taken advantage of in vivo. By generating local temperature changes, tissues can increase the malleability of liquid crystals and reform them to suit the desired function. The cortical EB liquid crystal tubules are always accompanied by Maltese-cross droplets and could be evidence of this supposition (Fig. 1).

Liquid crystals have long been found in the developing vital organs of a wide range of animals [4, 6, 26, 27]. Liquid crystals have also been reported in human vascular disease, liver dysfunction, age-related macular degeneration, and other chronic illnesses [28–36]. Given the pervasiveness of liquid crystals from embryogenesis to disease, identifying the function and underlying molecular mechanism of liquid crystals would lend great insight to a diverse set of cellular processes. However, the specific mechanism and function of liquid crystals during embryogenesis and illness remains unknown. This continuing mystery can be contributed to the complexity of embryonic development and the difficulties in simulating embryonic development in vivo.

Embryonic stem cells can differentiate into 250 cell-types required for constructing the entire repertoire of mammalian tissues and organs. Since all of these tissues develop after EB formation, the EB culture system is a model that allows researchers to investigate stem cell application in human diseases. Using this approach, we have unexpectedly discovered the existence of droplet and tubular liquid crystals in EB. These tubular complexes initiate at the embryoid body surface and are found throughout the cortex. Although most of the tubules remain near the cortical surface, some of the structures penetrate to the heart of the EB. Based on our results, we postulate that the formation of D/T-LC results in a three-dimensional “vascular” system that allows transport of nutrients and metabolic waste throughout the EB. Since a greater number of cells reside in the outer layers, this may explain the prevalence of LC tubules near the EB surface where more tubules are needed to ensure each cell receives adequate nutrients. However, the mechanisms for recognition and distribution of nutrients and waste through this D/T-LC system requires further exploration. The EB culture and differentiation approach developed here is an ideal in vitro model system for exploring liquid crystal function in embryogenesis and disease. Understanding of liquid crystal function in embryogenesis will not only shed light on a previously unknown aspect of development, but may also lead to pharmacological advances by unveiling new pathways through which we can penetrate tightly packed cell clusters, such as difficult to access tumor interiors.

## Additional file

**Additional file 1.** Additional information.

## Abbreviations

EBs: embryoid bodies
ES: embryonic stem
iPS: induced pluripotent stem
H&E: hematoxylin and eosin
CR: cortex region
D/T-LC: droplet and tubular liquid crystals
