# Supplementary material for: Characterization of tubular liquid crystal structure in embryonic stem cell derived embryoid bodies
Source: Cell Biosci. 2017 Jan 3;7:3. doi: 10.1186/s13578-016-0130-6 (PMC5210172; doi:10.1186/s13578-016-0130-6)

**Supplementary**

Source of Human Stem Cell

The human embryonic stem cell H9 and induced pluripotent stem cells (iPS DF19-9-7T) were purchased from WiCell Cytogenetics Laboratory, WiCell Research Institute WISC Bank, Wisconsin-Madison University.

Stem cell maintenance and differentiation

No-feeder culture system was used for stem cell maintenance. The human embryonic stem cell H9 and DF19-9-7T were maintained on culture plates coated with Matrigel (StemCell Technology, Madison, Wisconsin) in mTeSR-1 media with 10% Hyclone fetal calf serum at 37°C and 5% CO2 (StemCell Technology).

Total RNA of the stem cells was isolated from H9 and iPS DF19-9-7T cells using TRIzol reagent (Invitrogen) according to the manufacturer’s instructions. Reverse transcription reactions were performed with a SuperTranscript kit (Invitrogen) using random hexanucleotide primers and RNaseH-MMLV reverse transcriptase. Oct-3 expression in the embryoid bodies of human H9 and iPS DF19-9-7T cells was detected using RT-PCR with forwards primer (5’-GTTTTTGCTGCTGTGCCTGA-3’) and reverse primer (5’-GCATTGACACAGACAAGGTC-3’) (Figure S1) in addition to cytochemistry described below.

The human embryonic stem cell line H9 and iPS DF19-9-7T cells were used to carry out embryoid body aggregation. The ES cell clumps were cultured on low attachment Petri dishes coated with 0.1% gelatin. After cell clumps have aggregated, EBs were allowed to grow for 8 weeks and form differentiated spheres on low attachment Petri dishes. These EB differentiated spheres were then harvested for further experiments.

Immunocytochemistry analysis

After collection of embryoid bodies, samples were cryosectioned at a thickness of 10 -15 mm. The sections were rinsed five times with KSOM media to remove OCT, fixed in 3.7% formaldehyde in PBS for 10 minutes followed by neutralization in 50 mM NH4Cl in PBS for 10 minutes at room temperature. The samples were then permeabilized with 0.25% Triton X-100 in PBS for 10 minutes. Immunocytochemical staining was carried out by a two-hour incubation with primary Anti-Oct-3/4 antibody purchased from Transduction labs (Lexington, KY) in PBS/Tween (PBS containing 3% BSA and 0.1% Tween 20), followed by incubation with secondary antibody Peroxidase DAB HRP according to manufacturer’s protocol of Dako Envision-plus System (Dako North America, Inc., California). Stained samples were analyzed with a Zeiss Observer A1 microscope.

Polarization microscopy and phase transition analysis

After H9 and DF19-9-7T differentiated spheres were collected, the samples were prepared with smear-slide and cryosectioned in media with 20% glycerol PBS and observed under non-crossed polarizer and analyzer for conventional observations. Optical activity of the samples observed between crossed polarizer and analyzer were documented for further analysis. Observation on optical activity proceeded with XS-213A-P Polarization Microscope (Jnoec Ltd, Jiangnan, Nanjing, PRC) and Zeiss Observer.A1 microscope affiliated with Polarization accessory.

Measurements of H9 and DF19-9-7T differentiated spheres were performed on a combination of inverted microscope PE120 peltier system (Linkam Scientific Instruments, UK) and XS-213A-P polarization microscope. PE120 peltier system was set to work with 5mm aperture at heating-cooling rate 0.1 to 20°C/min ranged. Temperature stability is 0.1°C and controlled with RS232 on the temperature apparatus. Temperatures of phase transition between anisotropic and isotropic phases were recorded according to the observation of birefringence activities of the samples between two crossed polarizing prisms.

Pressure apply-and-release approach were utilized to characterize fluidity of birefringence in H9 and DF19-9-7T differentiated spheres. After samples were mounted between slide and cover-slips with PBS-glycerol media, pressure was applied to the cover-slip with a rubber applicator, and consequently released. Images of the entire process were captured continuously under polarization microscope and image analysis conducted.

H&E staining and histology

After collection of differentiated spheres derived from H9 and DF19-9-7T, samples were then cryosectioned at the thickness of 10 -15 mm. H&E staining were carried out as previously described for histological analysis [4, 6, 26, 27].

Image analysis and statistics

Images from conventional and polarization microscopy were captured with Zeiss Observer A1. The quantification of birefringent intensity was measured and constructed at different developmental stages with the image analysis software ImageJ 1.48v (NIH, Bethesda, MD). The data was analyzed with SPSS statistical software.

Supplementary Figure Legend

**Figure S1** Embryoid bodies were derived from human stem cells and induced pluripotent stem cells. Human stem cell H9 (panel A) and induced pluripotent stem cells (iPS DF19-9-7T, panel E) were used to aggregate into early embryoid bodies (panels B, C and F, G, respectively). Anti-Oct-3/4 antibody was used to detected pluripotency expression in H9 (panel D) and iPS DF19-9-7T (panel H) derived EBs with DAB Peroxidase detection. Stemness of undifferentiated EB were detected with Oct-3 expression (panel I). Scare bars are 200 mm in A, D, E and H; 600 mm in B, C, F and G.


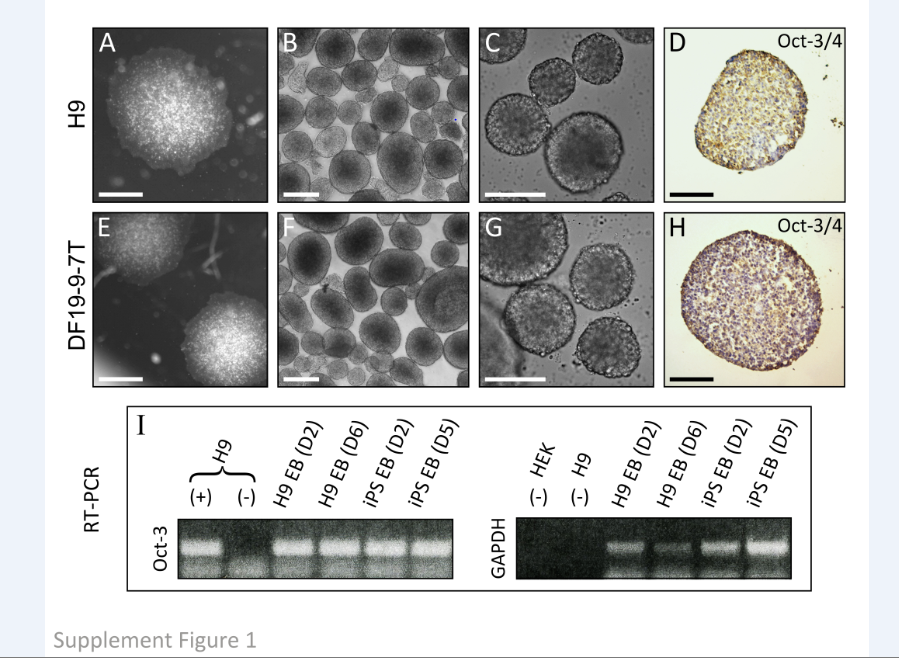

Supplement: Supplementary file 1 — Additional file 1. Additional information. [file 13578_2016_130_MOESM1_ESM.docx]
